# Morphological and molecular characterization of *Stomachicola muraenesocis* Yamaguti, 1934 (Digenea: Hemiuridae) from the daggertooth pike conger *Muraenesox cinereus* (Forsskål)

**DOI:** 10.1017/S0031182023001063

**Published:** 2024-01

**Authors:** Reza Ghanei-Motlagh, Jesús S. Hernández-Orts, Mark D. Fast, Shona K. Whyte, Mansour El-Matbouli, Mona Saleh

**Affiliations:** 1 Division of Fish Health, University of Veterinary Medicine, Vienna, Austria; 2 Hoplite Research Lab, Department of Pathology and Microbiology, Atlantic Veterinary College, University of Prince Edward Island, Charlottetown, PEI, Canada; 3 Natural History Museum, London, UK; 4 Institute of Parasitology, Biology Centre, Czech Academy of Sciences, České Budějovice, Czech Republic

**Keywords:** Dinurinae, Hemiuroidea, Lecithasteridae, molecular characterization, morphological variation, rDNA sequence, 18S, 28S, taxonomic revision, trematode

## Abstract

Hemiurid digeneans conspecific with *Stomachicola muraenesocis* Yamaguti, 1934 (the type species of the genus *Stomachicola* Yamaguti, 1934) were collected from the stomach of the daggertooth pike conger *Muraenesox cinereus* (Forsskål) off the Persian Gulf of Iran. This study aimed to provide a detailed characterization of *Stom. muraenesocis*, including measurements, illustrations and scanning electron microscopy (s.e.m.) representations. Comparisons with the original and previous descriptions revealed morphological and metrical variations in several features (i.e. body size and shape, arrangement of reproductive organs, soma to ecsoma length ratio, position of genital opening, number of vitelline tubules and extension of uterine coils) between *Stom. muraenesocis* from different hosts and localities. This study presents the first molecular sequence data associated with the small (18S) and large (28S) subunit nuclear ribosomal RNA genes (rDNA) for *Stom. muraenesocis*. Phylogenetic analyses of the 18S dataset placed *Stom. muraenesocis* as sister lineage to a clade formed of a group of species of *Lecithaster* Lühe, 1901 (Lecithasteridae Odhner, 1905). In contrast, phylogenetic analyses based on the 28S consistently recovered a sister relationship between *Stom. muraenesocis* and representatives of the Hemiuridae Looss, 1899. Further comprehensive phylogenetically based classification in light of morphology and taxonomic history of the Hemiuridae and Lecithasteridae is required to infer phylogenetic affinities and historical biogeography of *Stomachicola*. A comprehensive list of previously reported species of *Stomachicola* together with their associated hosts, localities and morphometric data is provided.

## Graphical abstract

**Figure:**
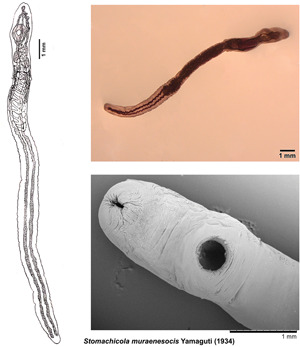

## Introduction

Members of *Stomachicola* Yamaguti, 1934 represent a group of digeneans included in the family Hemiuridae Looss, 1899 that are commonly found in the stomach of marine teleosts. Yamaguti (1934) erected the genus *Stomachicola* and recorded *Stomachicola muraenesocis* Yamaguti, 1934 as the type species from the stomach of the daggertooth pike conger *Muraenesox cinereus* (Forsskål) in the Inland Sea of Japan (South China Sea). *Stomachicola* was distinguished from *Dinurus* Looss, 1907 by having an unlobed seminal vesicle and a longer ecsoma (Yamaguti, 1934). Tseng (1935) reported representatives of *Stom. muraenesocis* collected from *M. cinereus* as *Lecithocladium longicaudum* Tseng, 1935. Some years later, Srivastava (1939) described *Stom. secundus* Srivastava, 1939 from the stomach of *Hyporhamphus limbatus* (Valenciennes), but this species was later transferred by Yamaguti (1958) to *Allostomachicola* Yamaguti, 1958. Bhalerao (1943) described specimens of *Stom. muraenesocis* from the Indian region and reported some morphological variations in the type species (number of vitelline tubes and extension of the uterus into the tail). Chauhan (1945) and Chauhan (1954) transferred *Lec. longicaudum* to *Stomachicola* and redescribed *Stom. muraenesocis* and *Stom. secundus* from *M. cinereus* and *Congresox talabonoides* (Bleeker) in India. Linton (1905) reported ovigerous and immature forms of *Distomum tornatum* Rudolphi, 1819 from several fish species inhabiting Beaufort, North Carolina, USA. Manter (1931) reevaluated Linton's materials and reidentified them as *Dinurus magnus* Manter, 1931. Later, Manter (1947) considered *Stomachicola* as a valid genus and transferred *Din. magnus* and *Din. rubeus* Linton, 1910 to *Stomachicola* (with the adjectival feminine names *Stom. magna* and *Stom. rubea*) based on the shape of the seminal vesicle. Skrjabin and Guschanskaja (1954) established *Pseudostomachicola* Skrjabin and Guschanskaja, 1954 and transferred *Stom. magna*, *Stom. rubea* and *Stom. secunda* to their newly erected genus based on the position of the genital pore and the distribution of vitellaria. Yamaguti (1958) erected the genus *Allostomachicola*, synonymized *Pseudostomachicola* with *Stomachicola* and placed *Stomachicola* and *Allostomachicola* in the subfamily Stomachicolinae (Yamaguti, 1958). He considered *Pseud. magna* and *Pseud. rubea* of Skrjabin and Guschanskaja (1954) as *Stom. magnus* and *Stom. rubeus*, and *Stom. secunda* of Srivastava (1939) as *Allo. secundus*. Sinclair *et al*. (1972) found no difference between the eggs of *Stom. magna* and *Stom. rubea* and considered *Stom. magna* as junior synonym of *Stom. rubea*.

Several species were later recorded in the literature as dissimilar types from *Stom. muraenesocis*. These include *Acerointestinecola karachiensis* Jahan, 1970, *Cameronia octovitellarii* Bilqees, 1971, *Cam. pakistani* Bilqees, 1971, *Cestodera gastrocecus* Bilqees, 1971, *Ces. unicecus* Bilqees, 1971, *Segmentatum karachiense* Bilqees, 1971, *Seg. qadrii* Bilqees, 1971, *Seg. cinereusis* Bilqees, 1971, *Seg. magnaesophagustum* Bilqees, 1971, *Indostomachicola kinnei* Gupta and Sharma, 1973, *Stom. mastacembeli* Verma, 1973, *Stom. lepturusi* Gupta and Gupta, 1974, *Stom. pelamysi* Gupta and Gupta, 1974, *Stom. polynemi* Gupta and Gupta, 1974, *Stom. bayagbonai* Siddiqi and Hafeezullah, 1975, *Linguastomachicola serpentina* Srivastava and Sahai, 1978, *Stom. singhi* Gupta and Ahmad, 1978, *Stom. rauschi* Gupta and Ahmad, 1978, *Stom. chauhani* Gupta and Singh, 1981, *Stom. chauhani* Pandey and Tewari, 1984 and *Stom. guptai* Gupta and Gupta, 1991. *Acerointestinecola* Jahan, 1970, *Cameronia* Bilqees, 1971, *Cestodera* Bilqees, 1971, *Segmentatum* Bilqees, 1971 and *Indostomachicola* Gupta and Sharma, 1973 were recognized congeneric with *Stomachicola* (Gibson and Bray, 1979; Hafeezullah, 1980). The 8 species described by Bilqees (1971) were transferred to the genus *Stomachicola* by Kazmi and Naushaba (2013); however, the authors were not aware that these species had been previously considered as junior synonyms of *Stom. muraenesocis* by Hafeezullah (1980). *Stomachicola mastacembeli*, *Stom. polynemi*, *Stom. singhi*, *Stom. bayagbonai*, *Stom. pelamysi*, *Lin. serpentina*, *Stom. chauhani* and *Stom. guptai* were also synonymized with *Stom. muraenesocis* (Hafeezullah, 1980, 1985; Gupta and Gupta, 1991; Tanzola and Seguel, 2012; Madhavi and Bray, 2018). Gupta and Singh (1981) transferred *Stom. lepturusi* and *Stom. rauschi* to *Allostomachicola* (Gupta and Singh, 1981; Gupta and Gupta, 1991; Tanzola and Seguel, 2012). Afterwards, 5 new species were described including *Stom. hainanensis* Shen, 1990, *Stom. sexaginta* Li and Sun, 1994, *Stom. pritchardae* Pande *et al*., 2000, *Stom. lycengraulidis* Tanzola and Seguel, 2012 and *Stom. bengalensis* Mishra *et al*., 2014.

The handling and processing of newly collected specimens of *Stomachicola*, in particular large worms, have been reported to be problematic primarily due to good development of musculature and contraction of the whole body of the parasite immediately after fixation (Sinclair *et al*., 1972; Hafeezullah, 1985). Moreover, several of the previous descriptions of species of *Stomachicola* were based on morphometric and morphological analyses performed by light microscopy using an inadequate number of specimens (Hafeezullah, 1985; Gupta and Gupta, 1991) or inadequate processing methods (e.g. different fixation temperatures, see Sinclair *et al*., 1972), which could influence some characteristics of taxonomic value. In addition, a high intraspecific (individual) variability in most morphological and metrical characters has been reported for *Stom. muraenesocis* (Bhalerao, 1943; Gupta and Gupta, 1991). Consequently, the validity of several species of *Stomachicola* has been questioned by several authors who consider them as synonyms of *Stom. muraenesocis* (Bhalerao, 1943; Hafeezullah, 1985; Gupta and Gupta, 1991; Madhavi and Bray, 2018).

Species of *Stomachicola* are characterized by possessing a long ecsoma, long filiform vitelline tubules and a sinuous pars prostatica (Madhavi and Bray, 2018). *Stomachicola* and *Allostomachicola* are distinguished by the position of the seminal vesicle (restricted to hindbody *vs* forebody) (Gibson and Bray, 1979). Species of *Stomachicola* are distributed worldwide and known to infect several fish species (particularly marine eels) from different families including Acanthuridae (Gupta and Ahmad, 1978; Pande *et al*., 2000), Anguillidae (Sinclair *et al*., 1972, Stunkard, 1980), Ariidae (Bilqees, 1971), Carangidae (Pandey and Tewari, 1984), Chirocentridae (Shen, 1990), Congridae (Gupta and Sharma, 1973; Gupta and Singh, 1981; Gupta and Gupta, 1991), Cynoglossidae (Corkum, 1966; Stunkard, 1973), Engraulidae (Tanzola and Seguel, 2012), Mastacembelidae (Verma, 1973), Megalopidae (Sinclair *et al*., 1972), Muraenesocidae (Yamaguti, 1934; Tseng, 1935; Bhalerao, 1943; Chauhan, 1945, 1954; Bilqees, 1971; Siddiqi and Hafeezullah, 1975; Srivastava and Sahai, 1978; Hafeezullah, 1980; Tang, 1981; Hafeezullah, 1985; Shen, 1990; Shen and Qiu, 1995; Hafeezullah and Dutta, 1998; Shaukat, 2008; Văn Hà *et al*., 2012), Muraenidae (Linton, 1910; Nahhas and Cable, 1964), Paralichthyidae (Corkum, 1966; Sinclair *et al*., 1972), Sciaenidae (Manter, 1931; Sinclair *et al*., 1972; Overstreet, 1983*a*, 1983*b*), Scombridae (Jahan, 1970; Gupta and Gupta, 1974), Serranidae (Nahhas and Short, 1965), Synbranchidae (Gupta and Gupta, 1991; Mishra *et al*., 2014) and Synodontidae (Linton, 1905; Manter, 1931; Corkum, 1959; Sinclair *et al*., 1972; Li and Sun, 1994).

In the present study, specimens of *Stom. muraenesocis* collected from the stomach of *M. cinereus* off the Persian Gulf of Iran were morphologically and morphometrically characterized using light and scanning electron microscopy (s.e.m.). For the first time, the phylogenetic affinities of *Stomachicola* within the superfamily Hemiuroidea Looss, 1899 were explored based on molecular sequence data from the small (18S) and large (28S) subunit nuclear ribosomal RNA genes (rDNA).

## Materials and methods

### Sample collection and preparation

A total of 30 daggertooth pike congers *M. cinereus* (total mean length ± standard deviation, 87.85 ± 6.50 cm) from Zir Ahak (28°17′N, 51°13′E), Bushehr, Iran, were examined between January and February 2021. The fish had been collected by fishing vessels along the coastal waters of the Persian Gulf before being purchased from local fishermen. The digestive tract of fresh fish was excised, placed in Petri dishes with saline and examined using a stereomicroscope. Specimens of *Stom. muraenesocis* were washed thoroughly in saline and relaxed following the procedure described by Dailey (1978). They were then killed with hot (nearly boiling) saline and fixed both in neutral-buffered formalin (10%) and in absolute ethanol. Some specimens were fixed in pure ethanol without being killed in hot saline for subsequent examination by s.e.m. (Cribb and Bray, 2010).

### Morphological examination

Digeneans were stained with Schneider's aceto-carmine solution (Gower, 1939), destained using acid ethanol, dehydrated in an ascending series of ethanol concentrations, cleared in glycerin and mounted in pure glycerin (semipermanent slides) or Canada balsam (permanent slides) (Wotton, 1937; Klimpel *et al*., 2019). Mounted specimens were measured using cellSens imaging software integrated with a digital camera (Olympus SC50 CMOS) installed on a compound microscope (Olympus BX-53). Line drawings were made with the aid of a drawing tube. All measurements are in millimetres and are presented as the range followed by the mean in parentheses. Parasite identification was performed according to the keys and descriptions provided by Yamaguti (1934), Yamaguti (1958), Gibson *et al*. (2002) and Madhavi and Bray (2018).

Infection parameters (i.e. prevalence, mean intensity and mean abundance) were calculated following Bush *et al*. (1997). To determine whether the ratios associated with soma length to width, soma to ecsoma length, oral sucker to pharynx length and oral sucker to ventral sucker length can be used as stable taxonomic characters, the range, mean ± standard deviation (s.d.) and coefficient of variation (CV, expressed in percent) of the ratios were calculated using the corresponding morphometric data obtained in this study and those reported in previous literature.

Two adult specimens preserved in pure ethanol were transferred into 70% acetone overnight and dehydrated in a series of increasing acetone concentrations. Then, they were treated with a mixture (1:1 v/v) of anhydrous acetone and hexamethyldisilazane (HMDS, Sigma–Aldrich, Germany) and immersed in HMDS (as the final desiccation step). Specimens were air-dried and mounted on metal stubs using conductive double-sided adhesive tape, coated with a thin layer of gold (4 nm) in a sputter coater (Balzers SCD 050) and examined with a tabletop scanning electron microscope (Hitachi TM-1000, operated at an accelerating voltage of 15 kV) equipped with a high-sensitive semiconductor BSE detector. Voucher specimens from the present study are deposited in the Parasite Collection of the Natural History Museum, London, UK (accession numbers 2023.2.27.6-7).

### Molecular identification

Two specimens of *Stom. muraenesocis* were separately placed into a pre-autoclaved laboratory mortar immersed in liquid nitrogen. As soon as most of the liquid nitrogen was evaporated, the trematodes were ground into a fine powder using an autoclaved pestle and placed in 2 mL Eppendorf tubes (Cox *et al*., 1990). Total genomic DNA was extracted from the homogeneous powders using the DNeasy Blood and Tissue kit in accordance with the manufacturer's guidelines (Qiagen GmbH, Hilden, Germany). PCR amplification of 28S rDNA gene was performed using the primers LSU5 and 1200R (Table 1) with the following cycling conditions: an initial denaturation at 95°C for 4 min, 35 cycles of denaturation at 95°C for 1 min, annealing at 55°C for 1 min, extension at 72°C for 1.5 min and a final extension step at 72°C for 5 min (Littlewood, 1994; Lockyer *et al*., 2003). The entire 18S rDNA gene was amplified by PCR using the primer sets Worm A and Worm B (Table 1) as described previously (Littlewood and Olson, 2001) with the following profile: an initial denaturation at 94°C for 2 min, followed by 40 cycles of 30 s at 94°C, 30 s at 54°C, 2 min at 72°C; and 7 min extension at 72°C. PCR reactions were carried out on a C1000 Touch Thermal Cycler (Bio-Rad) in a total volume of 50 μL containing 25 μL of DreamTaq Green PCR master mix (Thermo Scientific), 15 μL of nuclease-free water, 2 μL of 10 pmol μL^−1^ forward and reverse primers, 1 μL of 25 mm MgCl_2_ (Thermo Scientific) and 5 μL of 50 ng μL^−1^ DNA. PCR products were electrophoresed on 1% agarose gel (in Tris-acetate-EDTA buffer), excised from the gel and purified using a MinElute Gel Extraction Kit according to the manufacturer's instructions (Qiagen GmbH, Hilden, Germany). Purified DNA samples from 28S rDNA region were sequenced in both orientations using the same primers used in PCR reactions, while those from 18S rDNA region were sequenced using the 2 PCR primers and internal primers 1270R, 18SU467F and 18SL1170R (Table 1) (Littlewood and Olson, 2001; Indaryanto *et al*., 2015). Sequence data were generated using an automated sequencer (ABI 3730 XL) at LGC Biosearch™ Technologies (LGC Genomics GmbH, Berlin, Germany). Contiguous sequences were assembled manually, and base-calling were differences resolved using MEGA X and analysed with Chromas v2.6.6 to ensure accuracy (Sokolov *et al*., 2022). The newly generated sequences have been deposited in GenBank under accession numbers OR552105-OR552108.

**Table 1.** List of primers used in the present study

Molecular marker	Primer name	Sequence (5′–3′)	Reference
18S rDNA	Worm A	GCGAATGGCTCATTAAATCAG	Littlewood and Olson (2001); Indaryanto *et al*. (2015)
	Worm B	CTTGTTACGACTTTTACTTCC	
	1270R	CCGTCAATTCCTTTAAGT	
	18SU467F	ATCCAAGGAAGGCAGCAGGC	
	18SL1170R	GTGCCCTTCCGTCAATTCCT	
28S rDNA	LSU5	TAGGTCGACCCGCTGAAYTTAAGC	Littlewood (1994); Lockyer *et al*. (2003)
	1200R	GCATAGTTCACCATCTTTCGG	

### Phylogenetic analysis

The Basic Local Alignment Search Tool (BLAST) was employed to compare the novel sequences with publicly available sequences from GenBank. Newly generated sequences were aligned with those previously reported for species within the superfamily Hemiuroidea (see Table 2) following the alignments from Louvard *et al*. (2022*b*) and Pantoja and Kudlai (2022). Sequences of *Azygia longa* (Leidy, 1851) (Azygioidea: Azygiidae), *Proterometra* sp. (Azygioidea: Azygiidae), *Otodistomum cestoides* (Van Beneden, 1870) (Azygioidea: Azygiidae) and *Paucivitellosus fragilis* (Coil, Reid and Kuntz, 1965) (Bivesiculoidea: Bivesiculidae) were used as outgroups following Blair *et al*. (1998) and Sokolov *et al*. (2019). Only sequences with approximately similar lengths to our novel sequences were retrieved from GenBank. Sequences were aligned using ClustalW (Thompson *et al*., 1994) implemented in MEGA X (Kumar *et al*., 2018) with default parameters for the 18S dataset, and gap opening penalty and gap extension penalty values, respectively set at 15.00 and 5.00, for the 28S dataset (Sokolov *et al*., 2019), and the extremes of both alignments were trimmed to match the shortest sequences (Hall, 2013).

**Table 2.** Taxa included in the phylogenetic analyses with their host, locality, systematic position and GenBank accession number

Species	Definitive host	Locality	Family/subfamily	GenBank accession number		Reference
				28S rDNA	18S rDNA	
*Accacoelium contortum*	*Mola mola*	UK, Spain	Accacoeliidae/Accacoeliinae	AY222190	AJ287472	Olson *et al*. (2003); Ahuir-Baraja *et al*. (2015)
*Allogenarchopsis problematica*	—	Japan	Derogenidae/Halipeginae	MH628313	—	Sokolov *et al*. (2019)
*Annulocystis* cf. *auxis*	*Auxis thazard*	Australia	Didymozoidae/Gonapodasmiinae	OL336029	—	Louvard *et al*. (2022*b*)
*Annulocystis* sp.	*Auxis thazard*	Indonesia	Didymozoidae/Gonapodasmiinae	OL336031	—	Louvard *et al*. (2022*b*)
*Aphanurus mugilis*	*Osteomugil engeli*	Vietnam	Hemiuridae/Aphanurinae	LT607808; LT607809	LT607804; LT607805	Atopkin *et al*. (2017)
*Aponurus* sp.	*Mullus barbatus*, *Mullus surmuletus*	Spain	Lecithasteridae/Lecithasterinae	DQ354368; HQ713441	DQ354372	Pankov *et al*. (2006); Carreras-Aubets *et al*. (2011)
*Brachyphallus crenatus*	*Salvelinus leucomaenis*	USA	Hemiuridae/Lecithochiriinae	MH628299	—	Sokolov *et al*. (2019)
*Bunocotyle progenetica*	—	Spain	Bunocotylidae/Bunocotylinae	DQ354365	DQ354369	Pankov *et al*. (2006)
*Copiatestes filiferus*	*Trachurus murphyi*	New Zealand	Syncoeliidae/Syncoeliinae	AY222188	AJ287490	Cribb *et al*. (2001); Olson *et al*. (2003)
*Derogenes varicus*	*Hippoglossoides platessoides*	UK	Derogenidae/Derogeninae	AY222189	AJ287511	Cribb *et al*. (2001); Olson *et al*. (2003)
*Didymocylindrus* sp.	*Katsuwonus pelamis*	France	Didymozoidae/Didymozoinae	OL336001	—	Louvard *et al*. (2022*b*)
*Didymocystis* sp.	*Acanthocybium solandri*, *Auxis thazard*	Australia; Brazil	Didymozoidae/Didymozoinae	OL336003; OL336009; OP458335	—	Louvard *et al*. (2022*b*); Pantoja *et al*. (2022)
*Didymodiclinus* sp.	*Variola albimarginata*, *Diagramma labiosum*	Australia; Japan	Didymozoidae/Didymodiclininae	OL335999; OL336000	—	Louvard *et al*. (2022*b*)
*Didymozoid* sp.	*Taeniura lymma*	Australia	Didymozoidae/–	—	AY222102	Olson *et al*. (2003)
*Didymozoon scombri*	*Scomber scombrus*	UK	Didymozoidae/Didymozoinae	—	AJ287500	Cribb *et al*. (2001); Littlewood and Olson (2001)
*Didymozoon* sp.	*Cybiosarda elegans*	Australia	Didymozoidae/Didymozoinae	OL336015; OL336016; OL336017	—	Louvard *et al*. (2022*b*)
*Dinosoma synaphobranchi*	*Antimora microlepis*	Russia	Hemiuridae/Plerurinae	MH628303; MH628304	—	Sokolov *et al*. (2019)
*Dinurus longisinus*	*Coryphaena hippurus*	Jamaica	Hemiuridae/Dinurinae	AY222202	AJ287501	Cribb *et al*. (2001); Olson *et al*. (2003)
*Dinurus euthynni*	*Auxis thazard*	Brazil	Hemiuridae/Dinurinae	OP458333	—	Pantoja *et al*. (2022)
*Ectenurus virgula*	*Anisotremus virginicus*, *Decapterus punctatus*, *Prionotus punctatus*	Brazil	Hemiuridae/Dinurinae	OP918121; OP918122	—	Pantoja and Kudlai (2022)
*Elytrophalloides oatesi*	*Chaenocephalus aceratus*, *Notothenia coriiceps*, *Parachaenichthys charcoti*	Antarctic Peninsula	Hemiuridae/Elytrophallinae	ON123030	—	Faltýnková *et al*. (2022)
*Genarchella astyanactis*	*Astyanax* sp.	Nicaragua	Derogenidae/Halipeginae	OM502567	—	Santacruz *et al*. (2022)
*Genarchella pichileufue*	*Hatcheria macraei*	Argentina	Derogenidae/Halipeginae	LC630951	—	Tsuchida *et al*. (2021*b*)
*Genarchella* sp.	*Astyanax aeneus*, *Herichthys labridens*	Mexico	Derogenidae/Halipeginae	MK648276; MK648277	—	Pérez-Ponce de León and Hernández-Mena (2019)
*Genarchopsis chubuensis*	*Rhinogobius flumineus*	Japan	Derogenidae/Halipeginae	MH628311	—	Sokolov *et al*. (2019)
*Gonocerca crassa*	*Muraenolepis marmorata*	The Ross Sea	Gonocercidae/Gonocercinae	KY197012	—	Sokolov *et al*. (2018)
*Gonocerca muraenolepisi*	*Muraenolepis marmorata*	The Amundsen Sea	Gonocercidae/Gonocercinae	LN865025	—	Sokolov *et al*. (2018)
*Gonocerca oshoro*	*Albatrossia pectoralis*	The Sea of Okhotsk	Gonocercidae/Gonocercinae	KY197014	—	Sokolov *et al*. (2018)
*Gonocerca phycidis*	*Pogonophryne* sp.	The Ross Sea	Gonocercidae/Gonocercinae	KY197009	—	Sokolov *et al*. (2018)
*Gonocerca* sp.	*Muraenolepis marmorata*	The Ross Sea	Gonocercidae/Gonocercinae	HF543948	—	Sokolov *et al*. (2016)
*Gonocerca* sp.	*Muraenolepis marmorata*	The Ross Sea	Gonocercidae/Gonocercinae	LN650651	—	Sokolov *et al*. (2016)
*Genolinea anura*	*Pleurogrammus monopterygius*	Russia	Bunocotylidae/Opisthadeninae	MH628308	—	Sokolov *et al*. (2019)
*Genolinea bowersi*	*Chaenocephalus aceratus*, *Notothenia coriiceps*, *Parachaenichthys charcoti*	Antarctic Peninsula	Bunocotylidae/Opisthadeninae	ON123031	—	Faltýnková *et al*. (2022)
*Halipegus* sp.	*Lithobates* sp.	Mexico	Derogenidae/Halipeginae	MK648278	—	Pérez-Ponce de León and Hernández-Mena (2019)
*Helicodidymozoon tortor*	*Platycephalus speculator*	Australia	Didymozoidae/Nematobothriinae	OL336047	—	Louvard *et al*. (2022*b*)
*Hemipera manteri*	*Latridopsis forsteri*	Australia	Gonocercidae/Gonocercinae	AY222196	AY222105	Olson *et al*. (2003)
Hemiuridae gen. sp.	*Brycon guatemalensis*	Mexico	Hemiuridae/–	MK648287	—	Pérez-Ponce de León and Hernández-Mena (2019)
*Hemiurus levinseni*	—	Russia	Hemiuridae/Hemiurinae	MN962990; MN962997	—	Gonchar (unpublished)
*Hirudinella* sp.	*Makaira nigricans*, *Mulloidichthys martinicus*	Mexico	Hirudinellidae/–	KC985233	—	Calhoun *et al*. (2013)
*Hirudinella ventricosa*	*Euthynnus alletteratus*	Mexico	Hirudinellidae/–	MK648294	—	Pérez-Ponce de León and Hernández-Mena (2019)
*Hysterolecithoides guangdongensis*	*Siganus fuscescens*	China	Bunocotylidae/Hysterolecithinae	—	HM545901	Wang *et al*. (unpublished); Atopkin *et al*. (2018)
*Indodidymozoon* sp.	*Platycephalus endrachtensis*	Australia	Didymozoidae/Didymozoinae	OL336020	—	Louvard *et al*. (2022*b*)
*Isoparorchis eurytremus*	*Silurus asotus*	Japan	Isoparorchiidae/–	MH628315	—	Sokolov *et al*. (2019)
*Lampritrema miescheri*	*Brama japonica*	North Pacific Sea	Hirudinellidae/–	MW507472	—	Sokolov *et al*. (2021)
*Lobatozoum* sp.	*Thunnus albacares*	Australia	Didymozoidae/Didymozoinae	OL336021	—	Louvard *et al*. (2022*b*)
*Lecithaster confusus*	*Strongylura strongylura*, *Acanthogobius flavimanus*	Vietnam, Russia	Lecithasteridae/Lecithasterinae	MH625968; MH625975	—	Atopkin *et al*. (2018)
*Lecithaster gibbosus*	*Merlangius merlangus*	UK	Lecithasteridae/Lecithasterinae	AY222199	AJ287527	Cribb *et al*. (2001); Besprozvannykh *et al*. (2017)
*Lecithaster macrocotyle*	*Gymnodraco acuticeps*, *Parachaenichthys charcoti*	Ukraine	Lecithasteridae/Lecithasterinae	ON123032	—	Faltýnková *et al*. (2022)
*Lecithaster micropsi*	*Dissostichus mawsoni*, *Muraenolepis marmorata*	The Amundsen Sea, The Rose Sea	Lecithasteridae/Lecithasterinae	MH628306; MH628307	—	Sokolov *et al*. (2019)
*Lecithaster mugilis*	*Moolgarda seheli*, *Valamugil engeli*, *Liza subviridis*	Vietnam	Lecithasteridae/Lecithasterinae	LN865016; LN865021	LN865012	Besprozvannykh *et al*. (2017)
*Lecithaster salmonis*	*Cryptonatica affinis*	Russia	Lecithasteridae/Lecithasterinae	MH625980; OM850386	—	Atopkin *et al*. (2018); Krupenko *et al*. (2022*b*)
*Lecithaster sayori*	*Hemiramphus marginatus*	Vietnam	Lecithasteridae/Lecithasterinae	MH625977	—	Atopkin *et al*. (2018)
*Lecithaster* sp.	*Siganus fuscescens*	Vietnam	Lecithasteridae/Lecithasterinae	MH625978	—	Atopkin *et al*. (2018)
*Lecithaster sudzuhensis*	*Mugil cephalus*	Russia	Lecithasteridae/Lecithasterinae	LN865022; LN865023	LN865013	Besprozvannykh *et al*. (2017)
*Lecithochirium caesionis*	*Caesio cuning*	Australia	Hemiuridae/Lecithochiriinae	AY222200	AJ287528	Cribb *et al*. (2001); Olson *et al*. (2003)
*Lecithochirium* cf. *muraenae*	*Gymnothorax vicinus*	Brazil	Hemiuridae/Lecithochiriinae	OP918128	—	Pantoja and Kudlai (2022)
*Lecithochirium floridense*	*Percophis brasiliensis, Syacium papillosum*	Brazil, Mexico	Hemiuridae/Lecithochiriinae	MK558793; OP918131	—	Vidal-Martínez *et al*. (2019); Pantoja and Kudlai (2022)
*Lecithochirium microstomum*	*Prionotus punctatus, Trichiurus lepturus*	Brazil, Mexico	Hemiuridae/Lecithochiriinae	KC985235; OP918120; OP918127	—	Calhoun *et al*. (2013); Pantoja and Kudlai (2022)
*Lecithochirium* sp.	*Trichiurus lepturus*, *Octopus bimaculatus*	Mexico	Hemiuridae/Lecithochiriinae	MK648288; ON614673	—	Pérez-Ponce de León and Hernández-Mena (2019); Chan-Martin *et al*. (2022)
*Lecithochirium synodi*	*Anisotremus virginicus*, *Pseudopercis numida*	Brazil	Hemiuridae/Lecithochiriinae	OP918130; OP918132	—	Pantoja and Kudlai (2022)
*Lecithocladium excisum*	*Scomber scombrus*	UK	Hemiuridae/Elytrophallinae	AY222203	AJ287529	Cribb *et al*. (2001); Olson *et al*. (2003)
*Lecithophyllum botryophorum*	*Alepocephalus bairdii*, *Oneirodes thompsoni*, *Antimora microlepis*	UK, Russia	Lecithasteridae/Lecithasterinae	AY222205; MH628309	AY222107	Olson *et al*. (2003); Sokolov *et al*. (2019)
*Machidatrema chilostoma*	*Kyphosus vaigiensis*	France	Bunocotylidae/Hysterolecithinae	AY222197	AY222106	Olson *et al*. (2003)
*Merlucciotrema praeclarum*	*Cataetyx laticeps*	UK	Hemiuridae/Plerurinae	AY222204	AJ287535	Cribb *et al*. (2001); Olson *et al*. (2003)
*Metadidymozoon branchiale*	*Istiophorus platypterus*	Australia	Didymozoidae/Didymozoinae	OP793494	—	Louvard *et al*. (2022*a*)
*Myosaccium ecaude*	*Sardinella brasiliensis*	Brazil	Hemiuridae/Aphanurinae	OP918123	—	Pantoja and Kudlai (2022)
*Nematobothrium scombri*	*Scomberus scombrus*	UK	Didymozoidae/Nematobothriinae	AY222195	MG916943	Olson *et al*. (2003); Moreno (unpublished)
*Neodidymozoon* cf. *macrostoma*	*Istiompax indica*	Australia	Didymozoidae/Didymozoinae	OL336027	—	Louvard *et al*. (2022*b*)
*Neometadidymozoon elusivum*	*Platycephalus fuscus*	—	Didymozoidae/Didymozoinae	OL336025	—	Louvard *et al*. (2022*b*)
*Neometadidymozoon pterygionastes*	*Platycephalus indicus*	Australia	Didymozoidae/Didymozoinae	OL336028	—	Louvard *et al*. (2022*b*)
*Oesophagocystis* cf. *dissimilis*	*Katsuwonus pelamis*	French Polynesia	Didymozoidae/Didymozoinae	OL336022	—	Louvard *et al*. (2022*b*)
*Opisthadena dimidia*	*Kyphosus cinerascens*	Australia	Bunocotylidae/Opisthadeninae	AY222198	—	Olson *et al*. (2003)
*Opisthadena* sp.	*Kyphosus cinerascens*	Australia	Bunocotylidae/Opisthadeninae	—	AJ287549	Cribb *et al*. (2001)
*Paraccacladium* sp.	*Icichthys lockingtoni*	North-west Pacific	Paraccacladiidae/Paraccacladiinae	MW507467; MW507468	—	Sokolov *et al*. (2021)
*Parahemiurus merus*	*Harengula clupeola*, *Sardinella brasiliensis*	Brazil	Hemiuridae/Hemiurinae	OP918124; OP918125	—	Pantoja and Kudlai (2022)
*Plerurus digitatus*	*Scomberomorus commerson*	Australia	Hemiuridae/Plerurinae	AY222201	AJ287562	Cribb *et al*. (2001); Olson *et al*. (2003)
*Prosogonotrema bilabiatum*	*Caesio cuning*	Australia	Sclerodistomidae/Prosogonotrematinae	AY222191	AJ287565	Cribb *et al*. (2001)
*Progonus muelleri*	*Cryptonatica affinis*	Russia	Derogenidae/Derogeninae	OM761992	—	Krupenko *et al*. (2022*a*)
*Pulmovermis cyanovitellosus*	*Laticauda semifasciata*	Japan	Hemiuridae/Pulvoverminae	MH628314	—	Sokolov *et al*. (2019)
*Robinia aurata*	*Liza aurata*	Spain	Bunocotylidae/Bunocotylinae	DQ354367	DQ354371	Pankov *et al*. (2006)
*Saturnius gibsoni*	*Mugil cephalus*	Algeria	Bunocotylidae/Bunocotylinae	KJ010542	—	Marzoug *et al*. (2014)
*Saturnius* sp.	*Mugil cephalus*	Spain	Bunocotylidae/Bunocotylinae	DQ354366	DQ354370	Pankov *et al*. (2006)
*Stomachicola muraenesocis*	*Muraenesox cinereus*	Iran	Hemiuridae/Dinurinae	OR552107; OR552108	OR552105; OR552106	Present study
*Thometrema lotzi*	*Lepomis microlophus*	USA	Derogenidae/Halipeginae	KC985236	—	Calhoun *et al*. (2013)
*Thometrema patagonica*	*Percichthys trucha*	Argentina	Derogenidae/Halipeginae	LC586091	—	Tsuchida *et al*. (2021*a*)
*Tubulovesicula laticaudi*	*Hydrophis curtus*, *Hydrophis cyanocinctus*, *Hydrophis spiralis*	Sri Lanka	Hemiuridae/Mecoderinae	OR209733	—	Martin *et al*. (2023)
*Wedlia retrorbitalis*	*Thunnus obesus*	Australia	Didymozoidae/Koellikeriinae	OL336041	—	Louvard *et al*. (2022*b*)
*Outgroups*	—	—	—			
*Azygia longa*	*Esox niger*	USA	Azygiidae/Azygiinae	KC985234	—	Calhoun *et al*. (2013)
*Otodistomum cestoides*	*Raja montagui*	UK	Azygiidae/Azygiinae	AY222187	—	Olson *et al*. (2003)
*Proterometra* sp.	*Lepomis macrochirus*	USA	Azygiidae/Azygiinae	KC985237	—	Calhoun *et al*. (2013)
*Paucivitellosus fragilis*	*Crenimugil crenilabis*	Australia	Bivesiculidae/–	—	AJ287557	Cribb *et al*. (2001)

Phylogenetic trees were constructed using maximum likelihood (ML) and Bayesian inference (BI) analyses on XSEDE (Towns *et al*., 2014) using the CIPRES Science Gateway (Miller *et al*., 2010). Maximum likelihood analyses were performed using IQ-TREE v2.1.2 with 1000 bootstrap replicates (ultrafast bootstrap type) to estimate the nodal support (Minh *et al*., 2020). The best nucleotide substitution models GTR + F + I + G4 and TVM + F + R4 were respectively determined for 18S and 28S rDNA datasets in IQ-TREE under the Bayesian Information Criterion. Bayesian inference analyses were implemented using MrBayes v3.2.7a (Huelsenbeck and Ronquist, 2001). The best nucleotide substitution models (for BI analyses) were predicted with jModelTest v2.1.10 using the Akaike Information Criterion and Bayesian Information Criterion (Darriba *et al*., 2012). The best nucleotide substitution models GTR + I + G and TVM + I + G were estimated for the 18S and 28S rDNA sequence data, respectively (Pérez-Ponce de León *et al*., 2016). Bayesian analyses were performed using 2 independent 10 million generation runs of 4 simultaneous Markov chain Monte Carlo (MCMC) chains (nchains = 4) with trees sampled every 1000 generations (printfreq = 1000; samplefreq = 1000) and the first 3000 generations being discarded as burn-in (sump burnin = 3000; sumt burnin = 3000). Trees were re-rooted manually with designated outgroups and visualized using FigTree v1.4.4 (Rambaut, 2007).

## Results

### Taxonomic summary

#### *Stomachicola* Yamaguti, 1934

Syn. *Pseudostomachicola* (Skrjabin and Guschanskaja, 1954), *Acerointestinecola* (Jahan, 1970), *Cameronia* (Bilqees, 1971), *Cestodera* (Bilqees, 1971), *Segmentatum* (Bilqees, 1971), *Indostomachicola* (Gupta and Sharma, 1973) and *Linguastomachicola* (Srivastava and Sahai, 1978).

#### *Stomachicola muraenesocis* Yamaguti, 1934 (Figs 1–3)

Syn. *Distomum tornatum* of Linton, 1905, *Dinurus rubeus* (Linton, 1910), *Dinurus magnus* (Manter, 1931), *Lecithocladium longicaudum* (Tseng, 1935), *Stomachicola magna* (Manter, 1947), *Stomachicola rubea* (Manter, 1947), *Pseudostomachicola magna* (Skrjabin and Guschanskaja, 1954), *Pseudostomachicola rubea* (Skrjabin and Guschanskaja, 1954), *Acerointestinecola karachiensis* (Jahan, 1970), *Cameronia octovitellarii* (Bilqees, 1971), *Cameronia pakistani* (Bilqees, 1971), *Cestodera gastrocecus* (Bilqees, 1971), *Cestodera unicecus* (Bilqees, 1971), *Segmentatum karachiense* (Bilqees, 1971), *Segmentatum qadrii* (Bilqees, 1971), *Segmentatum cinereusis* (Bilqees, 1971), *Segmentatum magnaesophagustum* (Bilqees, 1971), *Indostomachicola kinnei* (Gupta and Sharma, 1973), *Stomachicola mastacembeli* (Verma, 1973), *Stomachicola polynemi* (Gupta and Gupta, 1974), *Stomachicola bayagbonai* (Siddiqi and Hafeezullah, 1975), *Stomachicola pelamysi* (Gupta and Gupta, 1974), *Stomachicola singhi* (Gupta and Ahmad, 1978), *Linguastomachicola serpentina* (Srivastava and Sahai, 1978), *Stomachicola chauhani* (Gupta and Singh, 1981), *Stomachicola chauhani* (Pandey and Tewari, 1984) and *Stomachicola guptai* (Gupta and Gupta, 1991).

**Figure 1.**
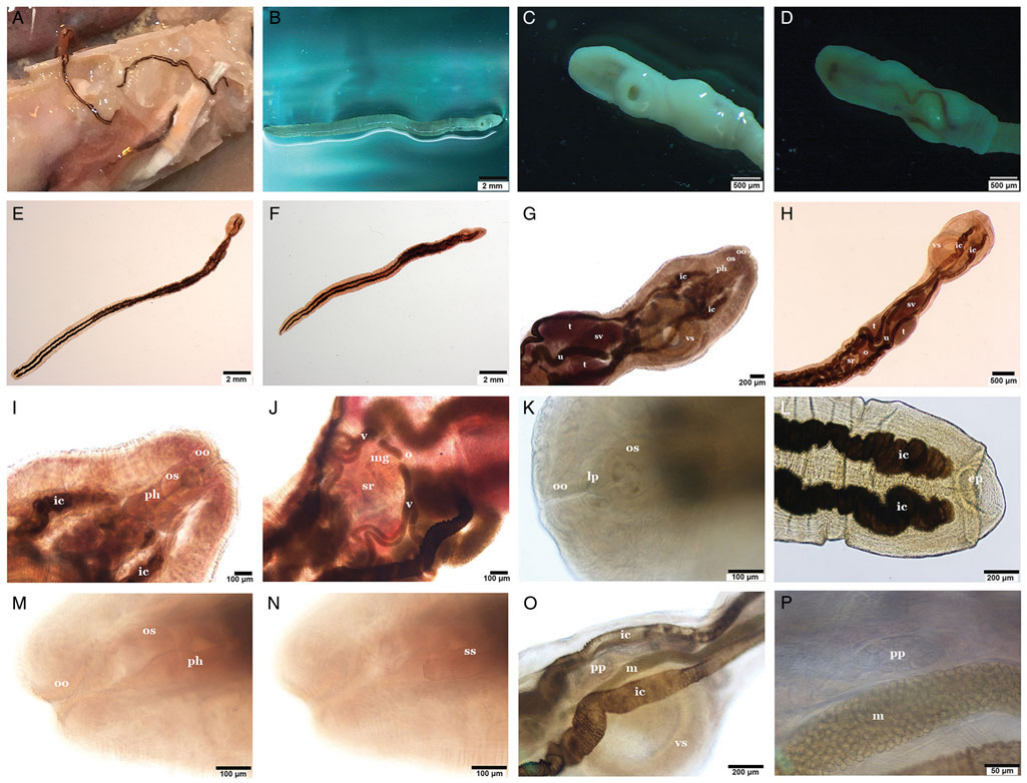
Microphotographs of the general morphology of *Stomachicola muraenesocis* from the stomach of *Muraenesox cinereus* from Zir Ahak, Bushehr, Iran. (A) Adult trematodes attached to the lumen of the stomach; (B) adult worm killed with hot saline, ventral view; (C) soma of an adult worm, ventral view; (D) soma of an adult worm, dorsal view; (E, F) ovigerous worms stained with Schneider's aceto-carmine, dorsal view; (G) soma of a stained adult worm, ventral view; (H) soma of a stained adult worm, dorsal view; (I) anterior end of an adult worm, ventral view; (J) detail of the female reproductive organs, ventral view; (K) ‘Linguiform projection’ arised from the oral sucker, ventral view; (L) posterior end of a stained adult worm, ventral view; (M) detail of the oral sucker, pharynx and oral sucker opening, ventral view; (N) detail of the sinus-sac, ventral view; (O) internal organs at level of ventral sucker, dorsal view and (P) detail of mature eggs inside the metraterm and large glandular cells of the pars prostatica. *Abbreviations:* ep, excretory pore; ic, intestinal caecum; lp, linguiform projection; m, metraterm; mg, Mehlis' gland; o, ovary; oo, opening of oral sucker; os, oral sucker; ph, pharynx; pp, pars prostatica; ss, sinus-sac; sv, seminal vesicle; t, testis; u, uterus; v, vitellaria; vs, ventral sucker.

**Figure 2.**
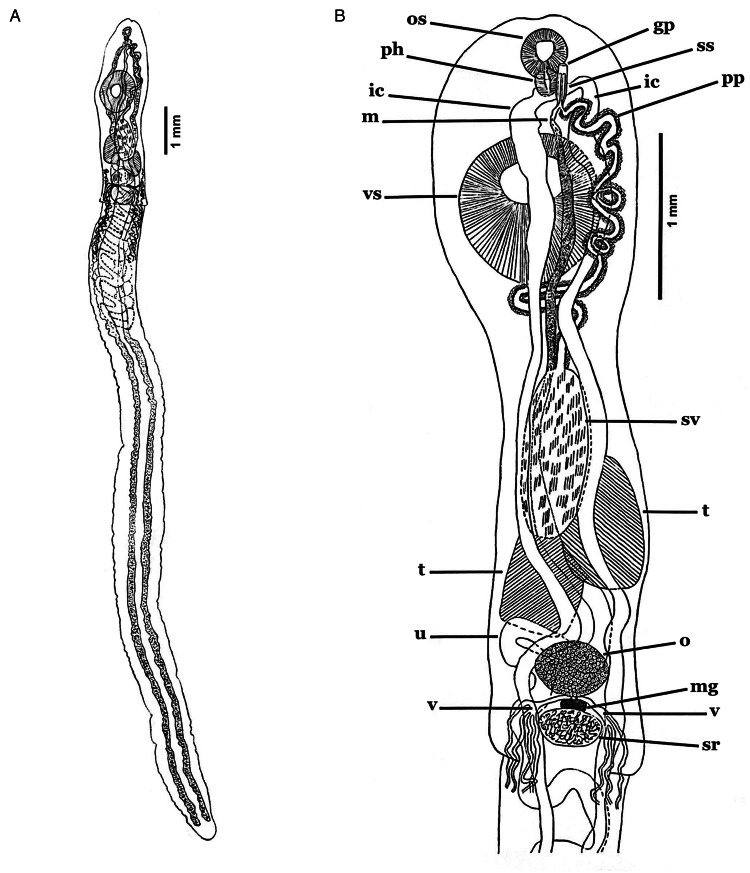
Line drawings of *Stomachicola muraenesocis* from *Muraenesox cinereus* from Zir Ahak, Bushehr, Iran. (A) Whole worm, ventral view and (B) soma, dorsal view. *Abbreviation:* gp, gential pore; ic, intestinal caecum; m, metraterm; mg, Mehlis' gland; o, ovary; os, oral sucker; ph, pharynx; pp, pars prostatica; ss, sinus-sac; sr, seminal receptacle; sv, seminal vesicle; t, testis; u, uterus; v, vitellaria; vs, ventral sucker.

**Figure 3.**
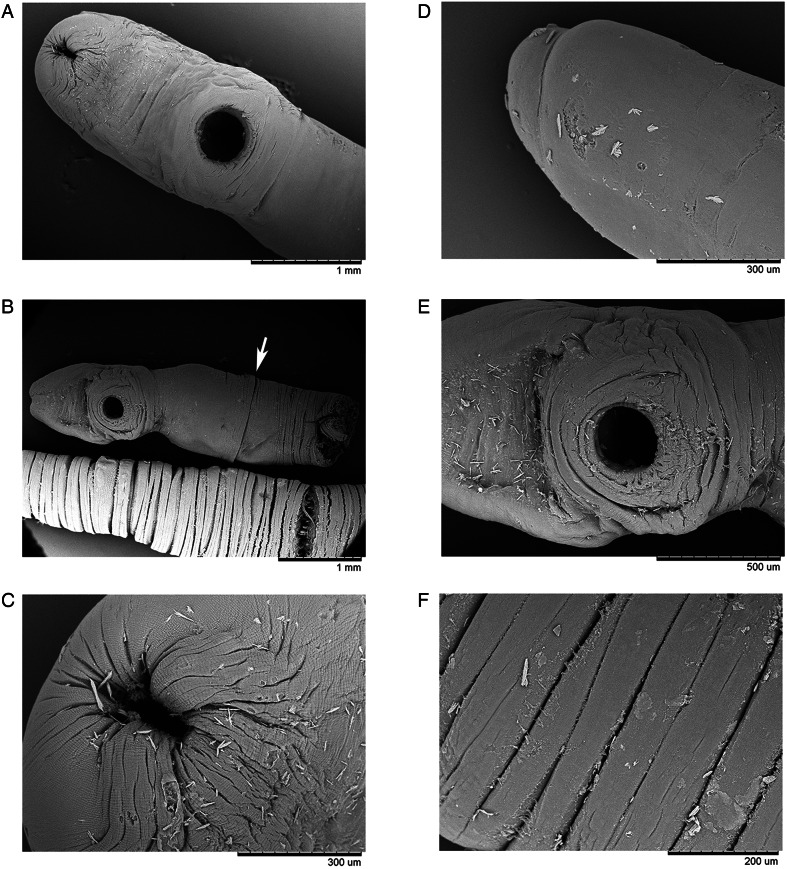
Scanning electron micrographs of *Stomachicola muraenesocis* from *Muraenesox cinereus* from Zir Ahak, Bushehr, Iran. (A) Soma, ventral view; (B) detail of the soma (ventral view) and the middle region of the ecsoma (ventral view), arrow points to the division between soma and ecsoma; (C) oral sucker, subapical view; (D) posterior end of the ecsoma, lateral view; (E) ventral sucker, ventral view and (F) detail of the ecsoma surface.

*Host:* Daggertooth pike conger, *Muraenesox cinereus* (Forsskål, 1775) (Anguilliformes: Muraenesocidae).

*Locality:* Zir Ahak (28°17′N, 51°13′E), Bushehr, Iran.

*Site of infection:* Stomach.

*Prevalence:* 43.3% (in 13 out of 30 fish).

*Mean intensity:* 18 worms.

*Mean abundance:* 7.8 worms.

*Total number of specimens collected:* 234 (213 ovigerous and 21 immature specimens).

### Description

**Figure 4.**
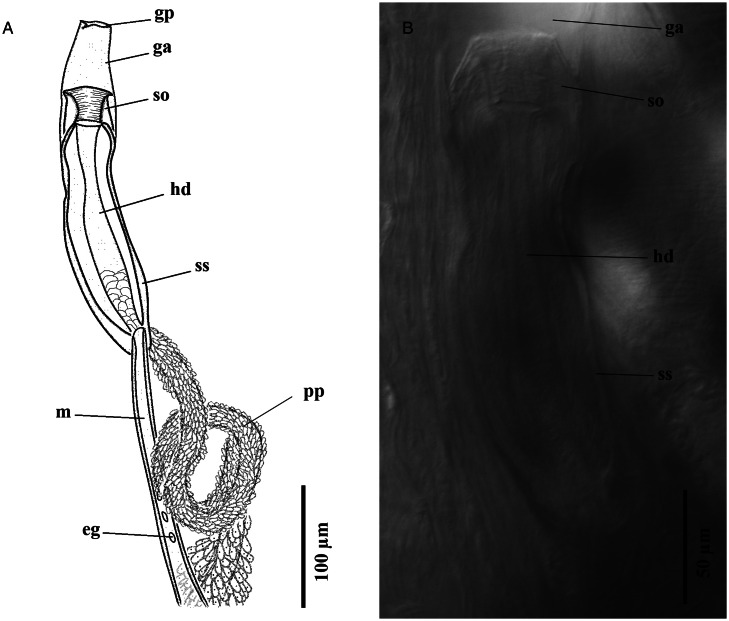
Terminal genitalia of *Stomachicola muraenesocis* from *Muraenesox cinereus* from Zir Ahak, Bushehr, Iran. (A) Line drawing of the terminal genitalia, ventral view and (B) microphotograph of the general morphology of the sinus-sac and sinus-organ, ventral view. *Abbreviation*: ga, genital atrium; gp, gential pore; eg, egg; hd, hermaphroditic duct; m, metraterm; pp, pars prostatica; so, sinus-organ; ss, sinus-sac.

Based on whole-mounts of 8 ovigerous adults and 2 specimens examined using s.e.m. (Figs 1–3). Measurements are presented in Table 3. Body (soma) elongated, curved ventrad (Figs 1C, 3A and B), cylindrical, widest at level of ventral sucker, narrower posterior to ventral sucker. Body surface smooth (Figs 1B–D and 3A). Body parenchyma refractive throughout (Figs 1B–D). Tegumental musculature is moderately thick and well-developed. Tegument is slightly to deeply segmented (Figs 1B and 3B), these segmentations do not truly exist because they are not observed in the live specimens. Ecsoma enormous, well developed (Figs 1B, E and F), dorsoventrally flattened. Soma and ecsoma are separated by telescoping demarcated line (Fig. 3B). Oral sucker is small, ventrally subterminal, spherical to subspherical or slightly funnel-shaped (Fig. 1I). Linguiform projection may arise from the lumen of oral sucker (Fig. 1K), connecting to the oral sucker opening near or precisely at the anterior extremity (Figs 1I and 3A). Preoral lobe short. Ventral sucker is large, rounded, and about 3 times larger than the oral sucker (Figs 1C, G, 3A and E). Forebody short relative to soma. Prepharynx absent. Pharynx globular to subglobular, slightly overlapping posterior border of oral sucker dorsally (Fig. 1I and M). Oesophagus very short. ‘Drüsenmagen’ present. Caeca is long, sinuous, filled with black-brown contents and terminates equally or unequally close to posterior end of ecsoma (Figs 1E, F and L). Testes pair, subtriangular to oval, symmetrical or oblique, almost at midlevel of hindbody (Fig. 1G and H). Seminal vesicle thin-walled, oval to elongate-oval, posterior to ventral sucker (Fig. 1G and H). Pars prostatica well-developed, undivided, tubular, convoluted, mostly or completely invested by large glandular cells (Fig. 1O and P), joins the base of the sinus-sac close to posterior margin of pharynx. Sinus-sac short, muscular and oval to pear-shaped (Figs 1N, 4A and B). Sinus-organ short, slightly muscular and permanent (Fig. 4A and B). Hermaphroditic duct short, straight, enclosed within sinus-sac and sinus-organ. Genital atrium short. Genital pore medial or slightly lateral, at the level of anterior pharynx or oral sucker, anterior to caecal bifurcation (Fig. 1N). Ovary reniform to oval, post-testicular or slightly overlapping testes (Fig. 1H and J). Mehlis' gland small, distinct, median and post-ovarian, between ovary and seminal receptacle (Fig. 1J). Juel's organ present. Laurer's canal was not observed. Seminal receptacle oval to transversely oval or irregularly round (Fig. 1J), medial, small to voluminous, at level of posterior soma or anterior ecsoma. Uterus coiled, mostly inter-caecal, usually extends up to two-thirds of the length of ecsoma and passes anteriorly dorsally to gonads and ventral sucker. Metraterm differentiated, thin-walled, joins male duct at sinus-sac base. Eggs are thick-walled, numerous, small and operculate (Fig. 1P). Vitelline lobes are tubular, mostly extra-caecal, formed by 2 main lateral tubes which are subsequently divided into 5–8, typically 7 (4 dextral and 3 sinistral or vice versa) slender tubes (Fig. 1J), extending from the posterior level of testes to anterior ecsoma. Excretory pore terminal on ecsoma. Excretory vesicle Y-shaped. Excretory arms united at the level of anterior soma.

**Table 3.** Comparative morphometric data for *Stomachicola* species from different fish hosts and localities

Species	*Dinurus rubeus*	*Dinurus magnus*	*Stomachicola muraenesocis*	*Lecithocladium longicaudum*	*Stomachicola muraenesocis*	*Stomachicola muraenesocis*	*Stomachicola muraenesocis*	*Acerointestinecola karachiensis*	*Cameronia octovitellarii*	*Cameronia pakistani*
Fish host	*Gymnothorax moringa*, *Gymnothorax funebris*	*Synodus foetens*, *Cynoscion nebulosus*	*Muraenesox cinereus*	*Muraenesox cinereus*	*Muraenesox cinereus*	*Muraenesox cinereus*	*Muraenesox cinereus*	*Cybium* sp.	*Muraenesox cinereus*	*Muraenesox cinereus*
Locality	USA	USA	Japan	China	India	India	Taiwan	Pakistan	Pakistan	Pakistan
Site of infection	Stomach, intestine	Stomach, swim bladder	Stomach	Stomach	Stomach	Stomach	Stomach	Intestine	Stomach	Stomach
Source	Linton (1910)	Manter (1931)	Yamaguti (1934)	Tseng (1935)	Bhalerao (1943)	Chauhan (1954)	Reid *et al*. (1966)	Jahan (1970)	Bilqees (1971)	Bilqees (1971)
Whole body (L × W^a^)	5.50–22.00 × 0.98–2.00	11–22 × 1.75–2.27	40.80 × 1.89	5.00–8.00 × –	49.00 × 2.70	55.00 × 2.50	18.10–31.70 × 0.50–0.78	10.00–43.00 × 1.05–1.87	—	—
Body proper (soma) (L × W)	− × 0.98–2.00	—	3.38^b^ × –	—	—	—	1.97–3.91 × 0.50–0.78	− × 1.05–1.87	6.00–7.98 × 2.43	4.00 × 2.30
Soma length to width ratio	—	—	—	—	—	—	1:0.20–0.25	—	1:0.30–0.40	1:0.57
Ecsoma (L × W)	—	—	37.42^b^ × –	—	—	—	14.9–27.80 × –	—	10.70–11.80 × –	16.80 × –
Soma to ecsoma length ratio	—	1:2.00	1:11.07	—	—	—	1:7.10–7.56	—	1:1.48–1.78	1:4.20
Oral sucker (L × W)	0.42–0.98 × 0.42–0.98	—	0.43 × 0.45	0.17–0.43 × 0.17–0.43	0.47 × 0.47	0.23–0.42 × 0.32–0.58	0.14–0.29 × 0.14–0.29	0.14–0.48 × 0.15–0.34	0.48 × 0.45	0.36 × 0.42
Pharynx (L × W)	0.28–0.42 × –	—	0.32 × 0.28	0.20–0.29 × 0.12–0.27	0.36 × 0.30	0.17–0.28 × 0.17–0.28	0.09–0.18 × 0.09–0.18	0.05–0.32 × –	0.24 × 0.27	0.27 × 0.24
Oral sucker to pharynx length ratio	1:0.43–0.67	—	1:0.74	1:0.67–1.18	1:0.76	1:0.67–0.74	1:0.62–0.64	1:0.36–0.67	1:0.50	1:0.75
Sinus-sac (L × W)	—	0.14 × –	—	—	0.40 × 0.23	—	—	—	—	0.40 × 0.16
Ventral sucker (L × W)	0.84–1.92 × 0.84–1.92	—	1.10 × 1.10	0.73–1.11 × 0.73–1.11	1.80 × 1.80	0.70–1.36 × 0.70–1.36	0.58–0.85 × 0.58–0.85	0.60–0.94 × 0.65–1.11	1.38 × 1.41	1.30 × 1.20
Sucker length ratio	1:1.96–2.00	1:2.00	1:2.40	1:2.58–4.29	1:3.83	1:3.04–3.23	1:2.93–4.14	1:1.96–4.28	1:2.87	1:3.61
Right testis (L × W)	—	—	0.44 × 0.48	0.36–0.46 × 0.35–0.45	0.53 × 0.80	0.46–0.84 × 0.53–0.64	0.29–0.44 × 0.25–0.38	0.29–0.32 × 0.29–0.32	0.39 × 0.42	0.42 × 0.36
Left testis (L × W)	—	—	0.45 × 0.37	0.36–0.46 × 0.35–0.45	0.33 × 0.42	0.28–0.46 × 0.52–0.74	0.29–0.44 × 0.25–0.38	0.29–0.32 × 0.29–0.32	0.42 × 0.45	0.30 × 0.36
Seminal vesicle (L × W)	—	—	0.63 × 0.50	0.67–1.07 × 0.41–0.53	0.72 × 0.54	—	0.56–0.78 × 0.30–0.43	0.71–0.78 × 0.49–0.51	0.69 × 0.24	0.24–0.36 × 0.21–0.48
Ovary (L × W)	—	—	0.26 × 0.77	0.08 × 0.18	0.40 × 1.22	0.31–0.46 × 0.53–0.85	0.26–0.40 × 0.45–0.50	0.60 × 0.73	0.48 × 1.08	0.36 × 1.05
Seminal receptacle (L × W)	—	—	0.42 × 0.47	0.97 × 0.67	0.50 × 0.40	—	—	—	0.84 × 1.26	0.42 × 1.20
Egg (μm) (L × W)	17–18 × 10	9–11 × 6–8	17–22 × 14	20 × 11	12–17 × 8–9	17–22 × 14	14–18 × 8–10	—	14 × 9	14 × 11
No. of vitellarian lobes	7	7	7	7	5, 7, 10	7	—	—	8	8

Measurements are in millimetres unless otherwise indicated.

a L × W: Range of length (L) × width (W) for each character is reported here (if specified by previous workers).

b These measurements were calculated based on the line drawing of the type species.

**Table:** 

Species	*Segmentatum karachiensis*	*Segmentatum qadrii*	*Segmentatum cinereusis*	*Segmentatum magnaesophagustum*	*Cestodera gastrocecus*	*Cestodera unicecus*	*Indostomachicola kinnei*	*Stomachicola pelamysi*	*Stomachicola mastacembeli*	*Stomachicola bayagbonai*	*Linguastomachicola serpentina*
Fish host	*Muraenesox cinereus*	*Muraenesox cinereus*	*Muraenesox cinereus*	*Muraenesox cinereus*	*Netuma thalassina*	*Netuma thalassina*	*Conger conger*	*Sarda chiliensis*	*Mastacembelus armatus*	*Cynoponticus ferox*	*Muraenesox talabonoides*
Locality	Pakistan	Pakistan	Pakistan	Pakistan	Pakistan	Pakistan	India	India	India	Nigeria	India
Site	Stomach	Stomach	Stomach	Stomach	Stomach	Stomach	Stomach	Stomach	Intestine	Stomach	Stomach
Source	Bilqees (1971)	Bilqees (1971)	Bilqees (1971)	Bilqees (1971)	Bilqees (1971)	Bilqees (1971)	Gupta and Sharma (1973)	Gupta and Gupta (1974)	Verma (1973)	Siddiqi and Hafeezullah (1975)	Srivastava and Sahai (1978)
Whole body (L × W)	—	—	—	—	31.00 × 2.60	65.00 × 2.50	—	34.80 × 2.35	3.80–23.34 × 0.50–1.80	9.44–21.15 × 2.13–3.99	− × 0.80–1.20
Body proper (L × W)	1.79–2.00 × –	4.40 × 2.60	4.07 × 2.03	3.86 × 1.27	6.00 × 2.60	5.50 × 2.50	5.25–7.50 × 1.45–2.02	− × 2.35	—	− × 2.13–3.99	2.90–4.10 × 0.80–1.20
Soma length to width ratio	—	1:0.59	1:0.50	1:0.33	1:0.43	1:0.45	1:0.27–0.28	—	—	—	1:0.27
Ecsoma (L × W)	10.10–12.90 × –	31.60 × –	37.00 × –	—	25.00 × –	59.00 × –	18.90–30.30 × –	—	—	—	—
Soma to ecsoma length ratio	1:5.64	1:7.18	1:9.10	—	1:4.17	1:10.73	1:3.60–4.04	—	—	—	—
Oral sucker (L × W)	0.16–0.18 × 0.30–0.32	0.40 × 0.50	0.50 × 0.40	0.18 × 0.29	− × 0.50	− × 1.2	0.15–0.27 × 0.16–0.28	0.25 × 0.30	0.14–0.24 × 0.14–0.27	0.28–0.52 × 0.36–0.61	0.19–0.31 × 0.19–0.31
Pharynx (L × W)	0.08–0.09 × 0.03–0.04	0.18 × 0.14	0.20 × 0.10	0.30 × 0.14	0.40 × 0.30	0.40 × 0.40	—	0.26 × 0.45	0.07–0.18 × 0.10–0.17	0.20–0.33 × 0.20–0.33	—
Oral sucker to pharynx length ratio	1:0.50	1:0.45	1:0.40	1:1.67	—	—	—	1:1.04	1:0.50–0.75	1:0.63–0.71	—
Sinus-sac (L × W)	—	—	—	—	—	—	—	—	—	—	—
Ventral sucker (L × W)	0.56–0.76 × 0.58–0.80	1.16 × 1.20	0.98 × 1.00	1.12 × 1.09	− × 2.10	− × 2.2	0.72–0.97 × 0.61–0.94	1.20 × 1.15	0.41–1.16 × 0.50–1.20	0.85–1.14 × 0.85–1.28	0.60–0.80 × 0.60–0.80
Sucker length ratio	1:3.50–4.22	1:2.90	1:1.96	1:6.22	—	—	1:4.20	1:4.80	1:2.92–4.83	1:2.40	1:2.58–3.16
Right testis (L × W)	0.17 × 0.20	0.60 × 0.65	0.60 × 0.50	0.10 × 0.25	0.40 × 0.50	—	0.52–0.67 × 0.55–0.60	0.44 × 0.26	0.30–0.45 × 0.24–0.43	0.42–1.02 × 0.38–0.76	0.36–0.58 × 0.29–0.25
Left testis (L × W)	0.18 × 0.21	0.60 × 0.58	0.70 × 0.40	0.10 × 0.21	0.45 × 0.56	—	0.54–0.60 × 0.48–0.54	0.35 × 0.46	0.28–0.48 × 0.21–0.43	0.42–1.02 × 0.38–0.76	0.27–0.56 × 0.25–0.27
Seminal vesicle (L × W)	0.11–0.14 × 0.12–0.16	0.40 × 0.76	0.20 × 0.14	—	0.81 × 0.90	0.45 × 0.81	1.06–1.23 × 0.58–0.73	0.85 × 0.60	0.37–0.79 × 0.19–0.59	—	—
Ovary (L × W)	0.17–0.19 × 0.18–0.20	0.60 × 0.50	0.30 × 0.70	0.36 × 0.70	0.45 × 0.90	0.51 × 1.27	0.43–0.51 × 0.72–0.82	0.80 × 0.57	0.10–0.46 × 0.59–0.79	0.33–0.52 × 0.54–0.67	0.23–0.27 × 0.36–0.40
Seminal receptacle (L × W)	0.45–0.46 × 0.51–0.52	0.50 × 1.10	0.80 × 0.90	0.29 × 0.36	—	1.05 × 1.10	Absent	0.40 × 0.25	Absent	—	—
Egg (μm) (L × W)	8 × 5	17 × 11	14 × 11	18 × 11	14 × 10	18 × 10	—	16–20 × 11–15	11–19 × 5–9	17–19 × 11–13	17–21 × 3–4
No. of vitellarian lobes	8	4	6	2	2	2	7	7	7	7	7

Species	*Stomachicola singhi*	*Stomachicola chauhani*	*Stomachicola muraenesocis*	*Stomachicola chauhani*	*Stomachicola hainanensis*	*Stomachicola muraenesocis*	*Stomachicola muraenesocis*	*Stomachicola sexaginta*	*Stomachicola pritchardae*	*Stomachicola muraenesocis*	*Stomachicola lycengraulidis*	*Stomachicola muraenesocis*	*Stomachicola muraenesocis*
Fish host	*Acanthurus triostegus*	*Uroconger lepturus*	*Muraenesox cinereus*	*Parastromateus* sp.	*Chirocentrus dorab*	*Muraenesox cinereus*	*Congromuraena anago, Ophisternon bengalense, Monopterus cuchia*	*Saurida elongata*	*Acanthurus triostegus*	*Muraenesox cinereus*	*Lycengraulis grossidens*, *Conger orbignianus*	*Congresox talabonoides*	*Muraenesox cinereus*
Locality	India	India	China	India	China	China	India	China	India	Pakistan	Argentina	Vietnam	Iran
Site	Stomach	Stomach	Stomach, esophagus	—	Stomach	Stomach	Stomach	Stomach, intestine	Stomach	Stomach	Stomach	Stomach	Stomach
Source	Gupta and Ahmad (1978)	Gupta and Singh (1981)	Tang (1981)	Pandey and Tewari (1984)	Shen (1990)	Shen (1990); Shen and Qiu (1995)	Gupta and Gupta (1991)	Li and Sun (1994)	Pande *et al*. (2000)	Shaukat (2008)	Tanzola and Seguel (2012)	Văn Hà *et al*. (2012)	Present study
Whole body (L × W)	5.04–15.81 × 0.75–0.82	39.30 × 2.58	14.70 × 1.60	30.00–39.00 × 1.25–2.40	9.12–44.42 × 0.77–2.47	14.70–39.34 × 1.39–3.29	6.84–15.10 × 0.62–0.93	14.60–23.10 × 1.34–1.63	32.00 × 2.34	21–21.51 × –	4.61–19.57 (11.56) × 0.94–2.35 (1.34)	16.75–17.25 × 0.72–0.86	8.46–35.67 (19.44) × 0.92–1.83 (1.20)
Body proper (L × W)	− × 0.75–0.82	15.70 × 2.58	4.30 × 1.60	− × 1.25–2.40	3.80–13.23 × 0.77–2.47	2.76–10.20 × 1.39–3.29	− × 0.62–0.93	3.80–4.50 × 1.34–1.63	9.80 × 2.34	4.30–4.50 × –	3.39–7.01 (4.86) × 0.94–2.35 (1.34)	—	2.97–7.13 (4.54) × 0.92–1.83 (1.20)
Soma length to width ratio	—	1:0.16	1:0.37	—	1:0.19–0.20	1:0.32–0.50	—	1:0.35–0.36	1:0.24	—	1:0.28–0.33 (0.27)	—	1:0.23–0.38 (0.27)
Ecsoma (L × W)	—	23.60 × –	10.40 × –	—	5.31–31.19 × –	—	3.39–12.44 × –	10.80–18.60 × –	22.20 × –	16.70–16.90 × –	1.22–12.56 (6.70) × –	—	5.49–28.54 (14.93) × 0.74–1.39 (1.07)
Soma to ecsoma length ratio	—	1:1.50	1:2.42	—	1:1.40–2.36	—	—	1:2.84–4.13	1:2.26	1:3.75–3.88	1:0.36–1.79 (1.38)	—	1:1.85–4.00 (3.16)
Oral sucker (L × W)	0.16–0.22 × 0.20–0.24	0.38 × 0.48	0.28 × 0.36	0.37–0.42 × 0.37–0.42	0.40–1.20 × 0.42–1.07	0.20–1.00 × 0.18–1.05	0.14–0.19 × 0.14–0.21	0.16–0.30 × 0.19–0.22	0.40 × 0.34	0.24–0.25 × 0.28–0.29	0.17–0.56 (0.31) × 0.22–0.46 (0.34)	0.18–0.19 × 0.22–0.25	0.23–0.44 (0.30) × 0.19–0.41 (0.29)
Pharynx (L × W)	0.12–0.14 × 0.11–0.13	0.24 × 0.25	0.19 × 0.17	0.22–0.25 × 0.22–0.25	0.25–0.72 × 0.25–0.72	0.12–0.58 × 0.12–0.62	0.08–0.12 × 0.08–0.13	0.12–0.16 × 0.11–0.15	0.29 × 0.25	0.29–0.30 × 0.30–0.31	0.10–0.25 (0.18) × 0.10–0.25 (0.17)	0.13–0.14 × 0.14–0.15	0.15–0.26 (0.20) × 0.08–0.23 (0.17)
Oral sucker to pharynx length ratio	1:0.64–0.75	1:0.63	1:0.68	1:0.68	1:0.6–0.62	1:0.58–0.60	1:0.53–0.57	1:0.53–0.75	1:0.72	1:1.20–1.21	1:0.45–0.59 (0.58)	1:0.72–0.74	1:0.59–0.65 (0.67)
Sinus-sac (L × W)	—	—	—	—	—	—	—	—	—	—	—	—	0.15–0.26 (0.21) × 0.05–0.10 (0.06)
Ventral sucker (L × W)	0.56–0.60 × 0.56–0.60	1.39 × 1.37	0.99 × 1.02	1.00–1.12 × 0.90–0.97	0.75–2.84 × 0.55–2.50	0.69–1.84 × 0.69–1.84	0.44–0.59 × 0.47–0.55	0.66–0.77 × 0.59–0.77	1.19 × 1.16	0.84–0.85 × 0.85	0.49–1.19 (0.82) × 0.47–1.29 (0.80)	0.58–0.66 × 0.54–0.63	0.75–1.02 (0.87) × 0.76–1.00 (0.87)
Sucker length ratio	1:2.72–3.50	1:3.66	1:3.53	1:2.67–2.70	1:1.87–2.37	1:1.84–3.45	1:2.50–3.36	1:2.57–4.12	1:2.97	1:2.90–3.00	1:1.71–2.89 (2.50)	1:2.40–2.52	1:2.12–3.70 (2.97)
Right testis (L × W)	0.48–0.55 × 0.34–0.47	0.60 × 0.59	0.44 × 0.22	0.52–0.58 × 0.37–0.45	0.40–0.83 × 0.23–0.67	0.37–0.65 × 0.27–0.71	0.32–0.54 × 0.15–0.49	0.24–0.40 × 0.33–0.59	0.62 × 0.50	0.53 × 0.30–0.31	0.26–0.63 (0.38) × 0.15–0.46 (0.29)	0.30 × 0.25	0.45–0.96 (0.69) × 0.32–0.62 (0.48)
Left testis (L × W)	0.45–0.58 × 0.34–0.42	0.43 × 0.35	0.23 × 0.22	0.52–0.58 × 0.37–0.45	0.43–1.20 × 0.33–0.64	0.33–0.67 × 0.25–0.78	0.30–0.45 × 0.19–0.32	0.21–0.52 × 0.31–0.47	0.49 × 0.49	0.50–0.35 × 0.33	0.24–0.45 (0.35) × 0.14–0.43 (0.30)	0.29 × 0.31	0.59–0.85 (0.71) × 0.35–0.61 (0.51)
Seminal vesicle (L × W)	0.47–0.65 × 0.29–0.44	0.46 × 0.56	0.44 × 0.54	1.00–1.11 × 0.67–0.75	0.45–1.17 × 0.13–0.50	0.50–1.37 × 0.50–1.50	0.34–0.67 × 0.10–0.49	0.67––0.84 × 0.46–0.59	0.75 × 0.45	0.74–0.75 × 0.35	0.22–1.16 (0.70) × 0.10–0.35 (0.23)	0.36–0.44 × 0.20–0.27	0.48–1.36 (0.71) × 0.30–0.48 (0.39)
Ovary (L × W)	0.11–0.25 × 0.17–0.39	0.69 × 1.12	0.34 × 0.85	0.12–0.15 × 0.14–0.16	0.27–0.68 × 0.33–1.42	0.12–0.53 × 0.20–1.04	0.17–0.49 × 0.14–0.31	0.34–0.45 × 0.67	1.02 × 0.85	0.24–0.25 × 0.21–0.25	0.10–0.46 (0.35) × 0.10–0.47 (0.28)	0.24 × 0.34	0.38–0.58 (0.44) × 0.43–0.89 (0.60)
Seminal receptacle (L × W)	Absent	1.10 × 1.16	—	—	0.17–0.50 × 0.17–0.83	0.08–1.30 × 0.17–1.00	—	0.38–0.71 × 0.47–0.92	0.40 × 0.30	—	—	—	0.18–0.67 (0.34) × 0.18–0.70 (0.32)
Egg (μm) (L × W)	15–20 × 10–12	19–20 × 9–11	—	7–15 × 6–10	15–18 × 9–12	15–18 × 6–12	7–21 × 5–11	17–20 × 11–14	12–13 × 9–10	5–8 × 5–7	13–18 (16) × 8–13 (11)	17–22 × 12–15	17–21 (19) × 9–21 (11)^a^
No. of vitellarian lobes	7	7	—	—	7	7	7	7	7	7	7	7	7

a Measurements obtained from 50 eggs.

### Molecular characterization and phylogenetic analysis

DNA fragments of different sizes were obtained for the 18S (1779–1789 nt) and 28S (980–982 nt) rDNA genes of *Stom. muraenesocis*. As sequences of *Stom. muraenesocis* are not available in GenBank, BLAST showed very low identity and low query coverage between the new sequences and the publicly available sequences of the 18S rDNA. However, our novel 28S rDNA sequences showed 94.51% identity and 99% query cover with an unidentified isolate (MK648287) of hemiurids (collected from a freshwater fish, *Brycon guatemalensis*) from Mexico (Pérez-Ponce de León and Hernández-Mena, 2019).

**Figure 5.**
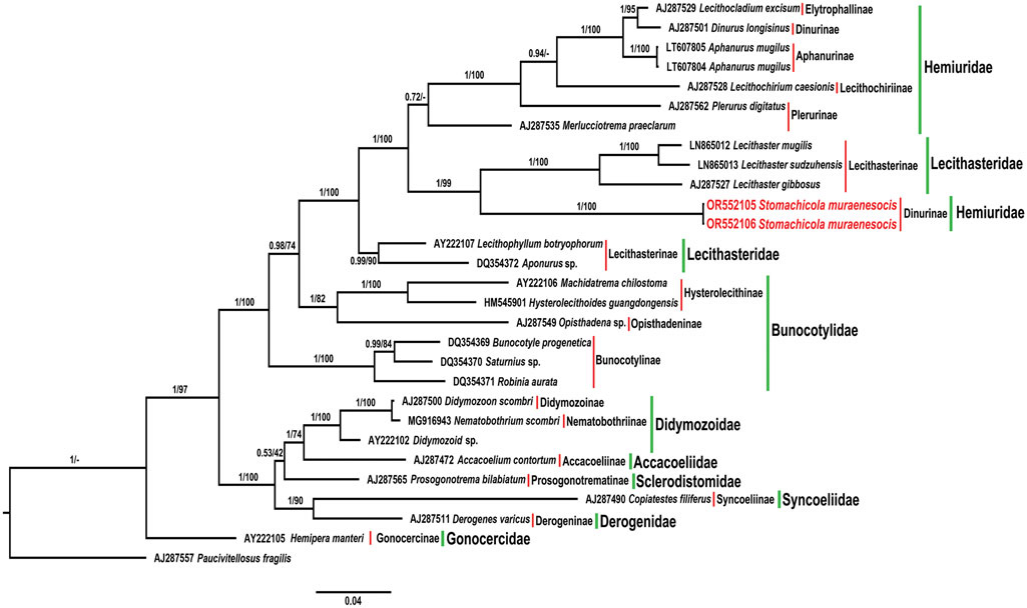
Bayesian inference phylogram reconstructed using the 18S rDNA sequences of *Stomachicola muraenesocis* (newly generated sequences are indicated in red color) and other members of the Hemiuroidea. The posterior probability and bootstrap support values are shown near the branches for Bayesian inference and maximum likelihood analyses, respectively. Red and green bars respectively represent different subfamilies and families to which taxa included in phylogenetic tree belong.

Maximum likelihood and BI trees obtained based on 18S rDNA sequences (Fig. 5) revealed the family Gonocercidae Skrjabin and Guschanskaja, 1955 as a basal group and the other families within the Hemiuroidea in 2 main clades with strong nodal support. The first clade includes representatives of the families Hemiuridae, Lecithasteridae Odhner, 1905 and Bunocotylidae Dollfus, 1950, whereas the second clade incorporates members of the families Didymozoidae Monticelli, 1888, Accacoeliidae Odhner, 1911, Sclerodistomidae Odhner, 1927, Syncoeliidae Looss, 1899 and Derogenidae Nicoll, 1910 (Fig. 5). Hemiuridae and Lecithasteridae formed a strongly supported clade in both trees, but none of these families was resolved as monophyletic. A surprising result of our phylogenetic analyses is that the sequences of *Stom. muraenesocis*, currently placed in the subfamily Dinurinae Looss, 1907 within the Hemiuridae on the basis of morphological characters, appeared with strong support as a sister to a clade formed by 3 *Lecithaster* species belonging to the Lecithasteridae (Fig. 5). This result shows that the position of *Stomachicola* within the Hemiuroidea needs to be reevaluated.

**Figure 6.**
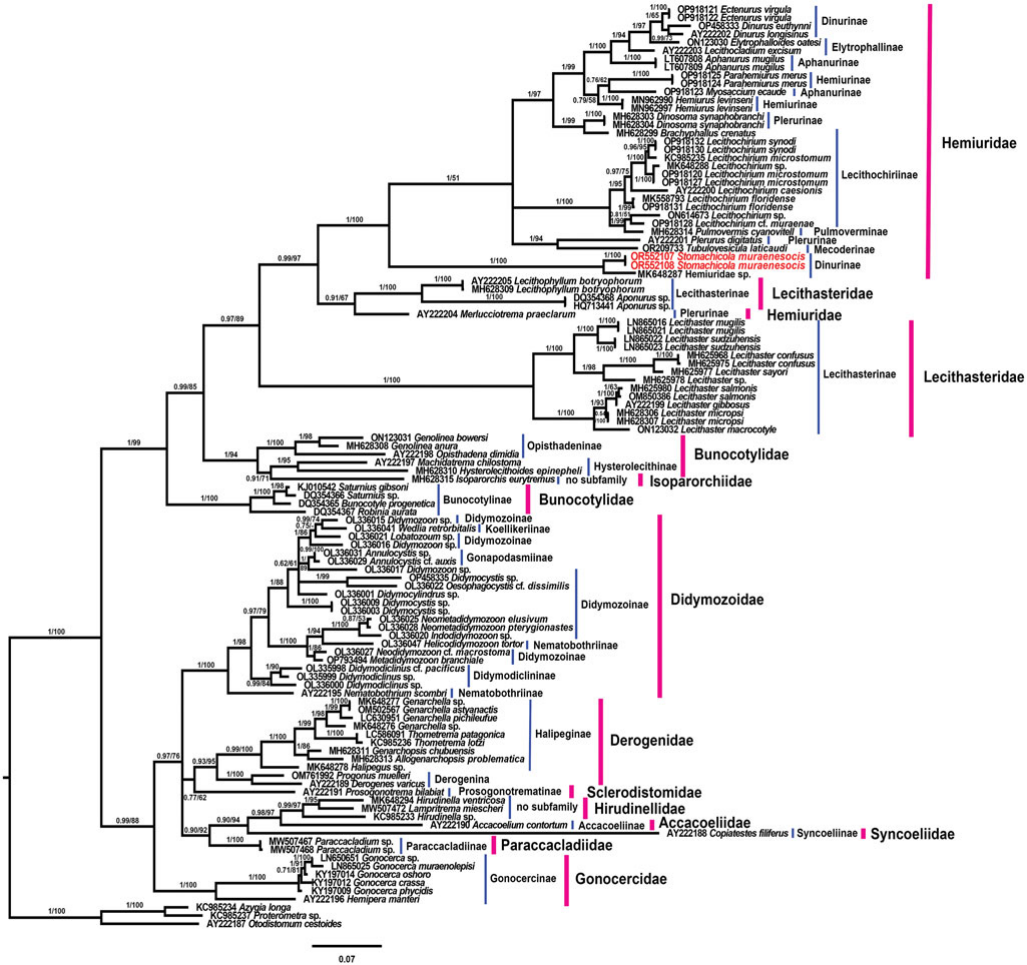
Bayesian inference phylogram reconstructed based on the 28S rDNA sequences of *Stomachicola muraenesocis* (newly generated sequences are indicated in red color) and other members of the Hemiuroidea. The posterior probability and bootstrap support values are given near the branches for Bayesian inference and maximum likelihood analyses, respectively. Blue and purple bars respectively indicate different subfamilies and families to which taxa included in phylogenetic tree belong.

Maximum likelihood and BI trees constructed from the 28S rDNA dataset (Fig. 6) yielded similar topologies. Our phylogenetic analyses resolved members of the Hemiuroidea into 2 distinct clades with strong support. Members of the Hemiuridae, Lecithasteridae, Bunocotylidae and Isoparorchiidae Travassos, 1922 formed a well-supported clade, sister to a clade composed of representatives of the families Didymozoidae, Derogenidae, Sclerodistomidae, Hirudinellidae Dollfus, 1932, Accacoeliidae, Syncoeliidae, Paraccacladiidae Bray and Gibson, 1977 and Gonocercidae with only strong posterior probability support. Phylogenetic analyses of the 28S rDNA dataset agreed with our phylogenetic assessment of the 18S rDNA dataset and resolved the Hemiuridae and Lecithasteridae as non-monophyletic families. Both ML and BI trees (28S rDNA data) recovered *Stom. muraenesocis* as sister to an undetermined hemiurid from Mexico (MK648287) in a strongly supported clade (Fig. 6). This clade constituted a sister group relationship with the remaining representatives (with the exception of *Merlucciotrema praeclarum*) of the Hemiuridae forming an ingroup polytomy of 3 clades in the BI tree (resolved as 2 distinct clades with moderate support in the ML tree). Furthermore, the monophyly of the subfamilies Lecithasterinae Odhner, 1905, Lecithochiriinae Lühe, 1901 and Plerurinae Gibson and Bray, 1979 was not supported.

## Discussion

The genus *Stomachicola* was proposed within the subfamily Dinurinae by Yamaguti (1934) to accommodate *Stom. muraenesocis* from the stomach of *M. cinereus* as the type species. Later, Yamaguti (1958) erected *Allostomachicola* and considered *Stomachicola* and *Allostomachicola* as members of the subfamily Stomachicolinae. The Stomachicolinae was synonymized with the Dinurinae by Gibson and Bray (1979), and this synonymy was accepted by Gibson *et al*. (2002) and Madhavi and Bray (2018). Martin *et al*. (2023) transferred the previously recognized dinurines without permanent sinus-organ to the Mecoderinae Skrjabin and Guschanskaja, 1954 (*Mecoderus* Manter, 1940, *Tubulovesicula* Yamaguti, 1934, *Stomachicola* and *Allostomachicola*). A total of 25 species of *Stomachicola* have been described to date. Of these species, 19 species are considered conspecific to *Stom. muraenesocis*, including *Stom. chauhani*, *Stom. guptai*, *Ind. kinnei*, *Stom. pelamysi*, *Stom. polynemi*, *Stom. rubea*, *Stom. magna*, *Lin. serpentina*, *Stom. mastacembeli*, *Stom. bayagbonai*, *Stom. singhi*, *Cam. octovitellarii*, *Cam. pakistani*, *Ces. gastrocecus*, *Ces. unicecus*, *Seg. karachiense*, *Seg. qadrii*, *Seg. cinereusis* and *Seg. magnaesophagustum* (Hafeezullah, 1980, 1985; Gupta and Gupta, 1991; Shaukat, 2008; Madhavi and Bray, 2018). Five species, i.e. *Stom. hainanensis*, *Stom. lycengraulidis*, *Stom. sexaginta*, *Stom. pritchardae* and *Stom. bengalensis* have not been revised so far and their systematic position and validity require further evaluation. There is currently no accepted key for the assigned species of *Stomachicola*, and the taxonomic status of some nominal species has never been verified.

Several morphological characters have been used to distinguish species of *Stomachicola*. For example, *Ind. kinnei*, *Stom. mastacembeli* and *Stom. singhi* were distinguished from other species by the absence of a seminal receptacle (Gupta and Sharma, 1973; Verma, 1973; Gupta and Ahmad, 1978). Nevertheless, the seminal receptacle could possibly be overlooked as it may contain no sperm in some specimens. In fact, the absence of a seminal receptable was not considered as a suitable character for species delimitation in *Stomachicola* by Hafeezullah (1980) and Hafeezullah (1985), who considered *Ind. kinnei*, *Stom. mastacembeli* and *Stom. singhi* as synonyms of *Stom. muraenesocis.* Pande *et al*. (2000) distinguished *Stom. pritchardae* from other species by having a tetra-lobed ovary. Since the ovary has been described as an unlobed organ in *Stomachicola*, the validity of such finding is required to be confirmed. *Stomachicola bayagbonai* is characterized by 2 frontal projections on either side of the preoral lobe (Siddiqi and Hafeezullah, 1975; Tanzola and Seguel, 2012). In the material examined in the present study; however, the morphology of the anterior part of the body and the appearance of the preoral lobe were found to vary between individuals of *Stom. muraenesocis* depending on the contraction state of the parasite during killing. In this regard, Sinclair *et al*. (1972) reported the appearance of the preoral lobe in *Stom. rubea* is highly influenced by the temperature of the fixative applied to living specimens. The presence of a ‘linguiform projection’ arising from the lumen of the oral sucker was used as a key feature to distinguish *Lin. serpentina* by Srivastava and Sahai (1978). The validity of the ‘linguiform projection’ to distinguish *Stomachicola* species was questioned by Hafeezullah (1980), and it was considered as a rare structure in this genus (Gibson *et al*., 2002). In the present study, ‘linguiform projection’ was observed in some specimens of *Stom. muraenesocis*, which suggests that this structure is rather variable. *Stomachicola bengalensis*, which was differentiated from *Stom. muraenesocis* by possessing an oral sucker larger than the ventral sucker, was proposed by Mishra *et al*. (2014), but the authors have never published the description of this species and thus it is invalid according to the International Code of Zoological Nomenclature (Ride, 1999). *Stomachicola hainanensis* collected from *Chirocentrus dorab* (Shen, 1990; Shen and Qiu, 1995) may be transferred to *Allostomachicola* by having a trilobed ovary and seminal vesicle located in the forebody.

*Stomachicola chauhani* of Pandey and Tewari (1984) was distinguished from *Stom. muraenesocis* mainly on the basis of the position of the genital pore (posterior to caecal bifurcation). *Stomachicola pelamysi* was also differentiated from *Stom. muraenesocis* by Gupta and Gupta (1974) due to the genital pore behind the caecal bifurcation and the diagonal testes. Previous observations showed that the position of the genital pore is anterior to the caecal bifurcation between the base of the oral sucker and the post-pharyngeal region in *Stom. muraenesocis* (Hafeezullah, 1980, 1985; Gupta and Gupta, 1991). In this study, the genital pore was observed medially or slightly laterally at the level of the oral sucker or pharynx. Therefore, the position of the genital pore should be used with caution for the identification of *Stomachicola* species. *Stomachicola chauhani* of Gupta and Singh (1981) was reported as a new species by having a demarcated line distant (twice) from the posterior end of seminal receptacle. On the other hand, Gupta and Gupta (1991) observed that the position of the genital pore, ventral sucker, testes, ovary and seminal vesicle, the extension of the uterus and vitellaria and the shape and size of internal organs are extremely variable among specimens of *Stom. muraenesocis*. Our findings also suggest the diverse morphological variations among individuals of the type species in terms of position, arrangement and size of reproductive organs. The number of vitelline lobes has been frequently used for distinction of species of *Stomachicola*. However, the number of these lobes may vary from 2 to 10 in different individuals. Moreover, the degree of expansion of the uterine coils entering the ecsoma was recognized as a largely varied feature among individuals (ranging from 32% to 71% of the total length). Tanzola and Seguel (2012) distinguished *Stom. lycengraulidis* from *Stom. muraenesocis* on the basis of the length of the ecsoma (25–70% of the total length in *Stom. lycengraulidis vs* about 92% of the total length in the type species reported by Yamaguti, 1934) and the development of the pars prostatica, seminal vesicle and hermaphroditic duct (highly developed *vs* less developed). However, it was found that the relative size of the ecsoma is considerably variable between species of *Stomachicola* (see below).

In this study, the ratios of soma length to width, soma to ecsoma length, oral sucker to pharynx length and oral sucker to ventral sucker length were calculated to predict possible stable taxonomic characters which could be used for description of species of *Stomachicola*. This allowed comparison of these ratios between all species of *Stomachicola* including those reported as *Stom. muraenesocis*, all species of *Stomachicola* excluding those reported as *Stom. muraenesocis* and only species reported as *Stom. muraenesocis*. The measurements of *Stomachicola hainanensis* were excluded from calculations because the species is morphologically associated with *Allostomachicola*. The range, mean ± s.d. and CV of the ratios for all species of *Stomachicola* were calculated as follows: soma length to width (*n* = 17, *n* represents the number of studies that reported the associated measurements) ranging from 1:0.16 to 1:0.59 with mean (±s.d.) 1:0.36 (±0.12) and CV 34.13%; soma to ecsoma length (*n* = 18) ranging from 1:0.36 to 1:11.07 with mean (±s.d.) 1:4.72 (±3.12) and CV 66.12%; oral sucker to pharynx length (*n* = 28) ranging from 1:0.36 to 1:1.67 with mean (±s.d.) 1:0.71 (±0.26) and CV 36.46%; oral sucker to ventral sucker length (*n* = 31) ranging from 1:1.71 to 1:6.22 with mean (±s.d.) 1:3.18 (±0.88) and CV 27.47%. The range, mean ± s.d. and CV of the ratios for all species of *Stomachicola* except the type species were as follows: soma length to width (*n* = 13) ranging from 1:0.16 to 1:0.59 with mean (±s.d.) 1:0.37 (±0.13) and CV 35.69%; soma to ecsoma length (*n* = 13) ranging from 1:0.36 to 1:10.73 with mean (±s.d.) 1:4.39 (±3.00) and CV 68.36%; oral sucker to pharynx length (*n* = 18) ranging from 1:0.36 to 1:1.67 with mean (±s.d.) 1:0.70 (±0.29) and CV 41.61%; oral sucker to ventral sucker length (*n* = 21) ranging from 1:1.71 to 1:6.22 with mean (±s.d.) 1:3.25 (±1.01) and CV 31.22%. The range, mean ± s.d. and CV of the ratios for species recorded as *Stom. muraenesocis* are summarized as follows: soma length to width (*n* = 4) ranging from 1:0.19 to 1:0.50 with mean (±s.d.) 1:0.32 (±0.08) and CV 29.94%; soma to ecsoma length (*n* = 5) ranging from 1:1.85 to 1:11.07 with mean (±s.d.) 1:5.56 (±3.61) and CV 65.01%; oral sucker to pharynx length (*n* = 10) ranging from 1:0.53 to 1:1.21 with mean (±s.d.) 1:0.73 (±0.18) and CV 24.94%; oral sucker to ventral sucker length (*n* = 10) ranging from 1:1.84 to 1:4.14 with mean (±s.d.) 1:3.04 (±0.48) and CV 15.69%. No specific value is considered low for a CV, but lower values of CV are correlated with less variability around the mean (Pélabon *et al*., 2020). Soma to ecsoma length ratio is therefore a variable and inappropriate distinguishing feature. The fact that the ratios calculated for soma length to width, oral sucker to pharynx length and oral sucker to ventral sucker length from all species (without the type species) closely overlap those from *Stom. muraenesocis* may suggest that the species previously reported are indeed the representatives of the type species. On the other hand, if the previous species are different from the type, these 3 ratios are not suitable for discrimination of different species belonging to the genus *Stomachicola*.

With respect to uncertainties related to previous synonymies proposed for species of *Stomatichola*, difficulties associated with acquisition of vouchers from different localities and morphological variations among individuals of the type species, it is practically impossible to provide a valid list of accepted species of the genus until detailed morphological and molecular studies have been carried out on material from a large number of hosts and localities. In the present study, the molecular sequence data associated with 2 popular genetic markers (18S and 28S) were obtained from *Stom. muraenesocis*, which will constitute the basis for future taxonomic studies of the genus *Stomatichola*. However, further nucleotide sequence data are required to demonstrate whether the previously recorded species from different hosts and localities represent different species, or they are genetically associated with the type species. Notably, analysis of sequence data associated with mitochondrial genetic markers may help to identify possible morphotypes of the type species that correspond to intraspecific morphological variations among individuals.

The 2 genera *Stomachicola* and *Allostomachicola* are characterized by a combination of common features such as muscular body, well-developed ecsoma, smooth tegument, muscular sinus sac, distinct–indistinct preoral lobe, tubular vitelline lobes (usually 7) and anteriorly united excretory arms as well as species-specific characters including the position of seminal vesicle (hindbody *vs* forebody) and type of pars prostatica (tubular *vs* vesicular) (Manter, 1940, 1947; Gibson and Bray, 1979; Gibson *et al*., 2002; Nahhas and Sey, 2002). Although the ecsoma is a variable characteristic in terms of development, *Stomachicola* and *Allostomachicola* can be differentiated from other ecsomate species within the Hemiuridae by possessing an extended ecsoma which is typically several times longer than the body proper. Based on gross morphology, *Stomachicola*/*Allostomachicola* belong to the Dinurinae (see Gibson and Bray, 1979). Recently, Martin *et al*. (2023) resurrected the Mecoderinae to accommodate the dinurines with a temporary sinus-organ (*Allostomachicola*, *Mecoderus*, *Stomachicola* and *Tubulovesicula*) and restricted the Dinurinae for dinurines representing a permanent sinus-organ (*Dinurus*, *Ectenurus*, *Erilepturus*, *Paradinurus* and *Qadriana*). Morphological examination of our specimens, however, revealed that the sinus-organ is of permanent type in *Stom. muraenesocis*. Permanent sinus-organ has been previously reported to be absent or rudimentary in *Stomachicola* (Gibson and Bray, 1979; Hafeezullah, 1985; Gibson *et al*., 2002; Madhavi and Bray, 2018). On the other hand, there are discrepancies in the literature about the presence of a permanent sinus-organ in *Allostomachicola* (Gibson and Bray, 1979; Hafeezullah, 1985). Moreover, the sinus-organ was found to be permanent and muscular in *Stom. lycengraulidis* (Tanzola and Seguel, 2012). According to Gibson and Bray (1979), the presence/absence and type of sinus-organ are mostly useful taxonomic features up to the subfamily level, and different types of sinus-organ (permanent and temporary) cannot occur in the same species of trematode. Therefore, such variability in the type of sinus-organ of the Dinurinae/Mecoderinae warrants further examination, and preparation of histological sections from the specimens is of utmost importance for definitive discrimination of the type of sinus-organ (Gibson and Bray, 1979).

Presently, the superfamily Hemiuroidea comprises 16 families among which molecular sequence data have been reported for certain members of the Accacoeliidae, Bunocotylidae, Derogenidae, Didymozoidae, Gonocercidae, Hemiuridae, Hirudinellidae, Isoparorchiidae, Lecithasteridae, Paraccacladiidae, Sclerodistomidae and Syncoeliidae. There are currently no sequences available for the species within the Bathycotylidae Dollfus, 1932, Dictysarcidae Skrjabin and Guschanskaja, 1955, Ptychogonimidae Dollfus, 1937 and Sclerodistomoididae Gibson and Bray, 1979. In the present study, the phylogenetic relationship of the representatives of the superfamily Hemiuroidea was not highly supported in the ML tree based on 28S rDNA dataset. However, the topologies obtained in the ML and BI trees were in general congruent with those obtained in previous studies (Pankov *et al*., 2006; Atopkin *et al*., 2017; Sokolov *et al*., 2019, 2021; Faltýnková *et al*., 2022; Pantoja and Kudlai, 2022; Louvard *et al*., 2022*b*). As the molecular data of members belonging to genera within the subfamilies Dinurinae/Mecoderinae (*Dinurus*, *Ectenurus* Looss, 1907, *Paradinurus* Vigueras, 1958, *Erilepturus* Woolcock, 1935, *Qadriana* Bilqees, 1971, *Allostomachicola*, *Mecoderus*, *Stomachicola*, *Tubulovesicula*) are largely unknown, only a few available sequences with similar length to those of *Stomachicola* were retrieved from the GenBank and included in phylogenetic analyses in this study. Bayesian inference and ML trees reconstructed based on 18S and 28S sequences illustrated that *Stomachicola* is not genetically clustered with the representatives of the subfamilies Dinurinae/Mecoderinae (*Dinurus longisinus* Looss, 1907, *Ectenurus virgula* Linton, 1910 and *Tubulovesicula laticaudi* Parukhin, 1969). Phylogenetic analyses based on the 18S rDNA region revealed the sister relationship between *Stomachicola* and *Lecithaster* in trees inferred by ML and BI models. The representatives of the genus *Lecithaster* are mainly found in the intestine of marine and euryhaline fish (Atopkin *et al*., 2018). The main difference between *Lecithaster* and *Stomachicola* is the presence of ecsoma in the latter, whereas the species of both genera represent the smooth body surface phenotype (Gibson *et al*., 2002). *Lecithaster* + *Stomachicola* constituted a distinct clade in both ML and BI trees, suggesting that the presence of ecsoma, which is a fundamental character for morphological differentiation of species within the family Hemiuridae, may not be associated with their molecular discrimination. In this regard, Atopkin *et al*. (2017) highlighted that texture of the body surface corresponds with molecular distinction of the subfamilies of Hemiuridae but the presence of ecsoma is not associated with taxonomic relationships of the representatives of the family. On the other hand, the basal position of *Stom. muraenesocis* + Hemiuridae gen. sp. (to the Hemiuridae group) on the phylogenetic trees reconstructed on the basis of 28S rDNA sequence data supports the possibility of the recognition of a distinct subfamily/family for representatives of the genus *Stomachicola*. However, determination of the exact subfamily/family to which *Stomachicola* belongs, from a molecular standpoint, requires further sequence data from closely related taxa.

## Data Availability

The datasets used and/or analysed are available from the corresponding author upon reasonable request. Nucleotide sequences of the 18S rDNA (OR552105-OR552106) and 28S rDNA (OR552107-OR552108) of *Stom. muraenesocis* have been deposited in GenBank.
